# Quality of life in home-dwelling cancer patients aged 80 years and older: a systematic review

**DOI:** 10.1186/s12955-022-02070-1

**Published:** 2022-11-28

**Authors:** Inger Helen Hardeland Hjelmeland, Jorunn Drageset, Øyvind Nordvik, Elisabeth Grov Beisland

**Affiliations:** 1 Faculty of Health and Social Sciences, Western Norway University of Applied Sciences, Stord, Norway; 2 VID Specialized University, Ulriksdal 10, 5009 Bergen, Norway; 3 Faculty of Health and Social Sciences, Western Norway University of Applied Sciences, Inndalsveien 28, 5063 Kronstad, Bergen, Norway; 4 Department of Global Health and Primary Care, University of Bergen, Postboks 7804, 5020 Bergen, Norway

**Keywords:** Quality of life, Instruments, 80 years and older, Home-dwelling cancer patients, Systematic review

## Abstract

**Objective:**

Quality of Life (QoL) in elderly cancer patients is a topic that has been little explored. This systematic review aims to identify, assess, and report the literature on QoL in home-dwelling cancer patients aged 80 years and older and what QoL instruments have been used.

**Methods:**

We systematically searched the databases of Medline, Embase, Cochrane Central Register of Controlled Trials (CENTRAL), PsykINFO, Scopus, Epistemonikos and Cinahl to identify studies of any design measuring QoL among home-dwelling cancer patients aged 80 years and older. We screened the titles and abstracts according to a predefined set of inclusion criteria. Data were systematically extracted into a predesigned data charting form, and descriptively analyzed. The included studies were assessed according to the Critical Appraisal Skills Programme (CASP) checklists, and the Preferred Reporting Items for Systematic Reviews and Meta-Analyses Statement (PRISMA) checklist was used to ensure rigor in conducting our investigations and reporting our findings. This systematic review was registered in PROSPERO (CRD42021240170).

**Results:**

We included three studies that specifically analyze QoL outcomes in the subgroup of home-dwelling cancer patients aged 80 years and older, with a total of 833 participants having various cancer diagnoses. 193 of the participants included in these three studies were aged 80 years or more. Different generic and cancer-specific QoL instruments as well as different aims and outcomes were studied. All three studies used a diagnosis-specific instrument, but none of them used an age-specific instrument. Despite heterogeneity in cancer diagnoses, instruments used, and outcomes studied, QoL in home-dwelling cancer patients aged over 80 years old seems to be correlated with age, physical function, comorbidity, living alone, needing at-home care services, being in a poor financial situation and having a small social network.

**Conclusion:**

Our systematic review revealed only three studies exploring QoL and its determinants in the specific subgroup of home-dwelling cancer patients aged 80 years and over. A gap in the knowledge base has been identified. Future studies of this increasingly important and challenging patient group must be emphasized. Subgroup analyses by age must be performed, and valid age and diagnosis specific QoL instruments must be used to generate evidence in this segment of the population.

**Supplementary Information:**

The online version contains supplementary material available at 10.1186/s12955-022-02070-1.

## Introduction

The fraction of the population aged over 80 years old is rapidly increasing worldwide and the numbers are expected to further increase. According to estimates, the population of people aged over 80 years old is expected to rise to 60.8 million in the EU 27 by 2100 [1]. As a person’s age increases, so too it seems, does their morbidity, and cancer is particularly prevalent among older age groups. More than 75% of people with cancer report at least one other condition, and multimorbidity (defined as the co-existence of two or more conditions) increases with age [2]. People aged 80 and older have significantly more stays in hospital, and are generally more in need of home care services than the younger segment of the population [3, 4]. Cancer patients aged 80 or older represent a population who are vulnerable and frail. They may have multiple health issues and comorbidities, and cancer treatment can be challenging and complex [5, 6]. Comorbidity and cancer treatment can affect how the elderly patients experience their Quality of Life (QoL) [3, 7]. The use of a gold standard geriatric evaluation tool, the Comprehensive Geriatric Assessment (CGA) shows that impairment in geriatric populations is related to reduced QoL [8]. Several studies show that repeated testing of QoL during the course of treatment can be valuable as a tool for tailoring treatment and as a predictor of survival [7]. Differences in assessment of symptoms and QoL between home-dwelling cancer patients and their specialist palliative care nurse, underlines the significance and necessity of the patient’s own perspective in the assessment of both aspects [9].

The World Health Organization (WHO) defines quality of life as “*an individual’s perception of their position in life, in the context of the culture in which they live and in relation to their goals, expectations, standards and concerns*” [10]. The term QoL is currently often used synonymously with the term health related quality of life (HRQoL). Padilla et al. [11] states that HRQoL can be defined as “*a personal, evaluative statement summarizing the positivity or negativity of attributes that characterize one’s psychological, physical and social functioning, and spiritual well-being at a point in time when health, illness, and treatment conditions are relevant*”. QoL and HRQoL are measured by either generic or disease-specific questionnaires, resulting in a score [12]. Most QoL instruments developed over the past 10 years reflect elements of the approach advocated by Padilla et al. [10, 11].

A cancer diagnosis and frailty were associated with worse HRQoL both at baseline and at follow-up in a study of community-dwelling people aged 65 and over both with and without a cancer diagnosis [13]. A study focusing on older people (mean age 77.4 years) with a cancer diagnosis living at home and needing assistance from home nursing services showed a significant difference between men and women in terms of marital status, ongoing treatment, anxiety and depression, as well as an association between pain, fatigue and anxiety [14]. The complexity of symptom burden, both physical and psychological, should be emphasized when assessing older cancer patients. Individual-oriented and pro-active care for multimorbid elderly patients can reduce the risk of high-level emergency care, increase the use of low-level planned care, and substantially reduce mortality risk [15]. Appropriate treatment of cancer related problems can improve QoL [4]. However, little is known on how elderly cancer patients experience their QoL, as they seem to be insufficiently included in clinical trials as age limits are often used as exclusion criteria [3]. A recently published systematic review found no direct evidence regarding health and quality of life (QoL) in cognitively intact *hospitalized* cancer patients aged over 80 years old [16]. This study was conducted in a different setting, but still reflects the lack of evidence in the field of cancer patients aged 80 years and older. An important finding in this review was that none of the studies used an age- specific instrument [16].. According to the “COSMIN Study Design checklist for Patient-reported outcome measurement instruments” [17] an important criterion when measuring a selected sample, such as elderly cancer patients, is that the selected sample should represent the target population in which the PROM is validated, in terms of age, gender, and important disease characteristics.

To the best of our knowledge, a systematic review aiming to report on QoL in home dwelling cancer patients more than 80 years of age, has not yet been published. There is an urgent need to generate insight and establish evidence on QoL from the perspective of this continuously growing segment of the population, and what instruments have been used.

### Aim

The aim of this systematic review is to present how QoL is reported in studies of home-dwelling cancer patients aged 80 years and older, and what QoL instruments have been used.

## Methods

The implementation and reporting of this review has been performed according to the Preferred Reporting Items for Systematic Reviews and Meta-analyses (PRISMA) guidelines [18]. A data charting/extraction form was adapted from “The PRISMA 2020 statement: an updated guideline for reporting systematic reviews” BMJ 2021 [19]. The review was registered in PROSPERO (CRD42021240170) [20].

### Eligibility criteria

We included primary studies of any design reporting on quality of life (QoL) and/or health-related QoL (HRQoL), using validated questionnaires, in home-dwelling cancer patients aged over 80 years old. Studies were included if they met the following a priori eligibility criteria: (1) home-dwelling people with any cancer diagnosis, (2) included a measurement of QoL/HRQoL, (3) participants aged over 80 years old with data analyses by age group, (4) the number of cancer patients aged 80 or older included in the study was stated, and (5) the abstract language being in English.

### Search strategy

Studies were identified through structured searches of all publication years (final update search performed 20.09.2021) in the following electronic databases: Medline via OVID, Embase via OVID, Cochrane Central Register of Controlled Trials (CENTRAL), PsykINFO, Scopus, Epistemonikos and Cinahl via EBSCO. The search strategy was developed in consultation with a specialist librarian at the Western Norway University of Applied Sciences. Medical Subject Headings (MeSH) terms in Medline were developed to search for all key concepts and were modified for other databases. To combine the terms, Boolean logic was used. Keyword searches restricted to abstract and title were also conducted in the different databases (Additional file 1 Appendix 1). The process of inclusion proceeds by the PRISMA Flow diagram (Additional file 1 Appendix 2).

### Screening and study selection

All identified articles were uploaded into an EndNote X8 database and duplicates were removed. Then all remaining articles, 4,419, were exported to the software Rayyan (https://www.rayyan.ai/) for screening. Preliminary screening was undertaken by the university librarian to remove obvious exclusions (e.g. conference abstracts, etc.) after which all four authors (IHHH, EGB, ØN and JD) independently screened half of the articles each against eligibility criteria taking title, abstract and full text into account. Disagreements were discussed and resolved by consensus in the pairs. Any unresolved items were reviewed by an independent third author and the decision stood. If criteria were unclear in the manuscript, corresponding authors were consulted and asked for clarification.

### Quality assessment

Two authors (JD and ØN) assessed the quality of the included articles by using the Critical Appraisal Skills Programme (CASP) checklists [21] adapted to the study designs cohort and cross-sectional designs. Critical assessment was graded 1 point for “Yes", 0.5 points for “Can’t tell” (unsure), and 0 points for “No” according to the guidelines put forward by Butler et al. [22].

### Data extraction and outcomes

A data extraction form was developed and trialed by the research team to extract data about study details and characteristics (e.g. country, sample characteristics, QoL instruments used, outcomes, key findings and authors conclusion). Two articles were then randomly selected, and all reviewers reviewed the data extracted. As there were no discrepancies, data extraction by a second reviewer for the remaining articles was considered unnecessary. Data were systematically extracted into the predesigned data charting form, and descriptively analyzed. To describe the nature of the studies targeting QoL/HRQoL in home-dwelling older adults aged over 80 years old with cancer, we extracted detailed information related to QoL or HRQoL and the validated instruments measuring these outcomes. The primary outcomes for each study were identified and noted.

## Results

We included three studies that had home-dwelling cancer patients aged 80 and older in their cohorts, and which presented estimates of QoL/HRQoL in this subgroup (Table 1) [23–25].

**Table 1:** Results and characteristics of the included studies

nr	Author, (year), Country	Aim	Design	Participants/control group	QoL instruments used	Results	Authors conclusion	a) CASP score
1	Krahn et al., 2013, Canada [23]	To measure quality of life (QoL) and utilities for prostate cancer (PC) patients and determine their predictors	Prospective cohort study	A population-based, community-dwelling, geographically diverse sample of long-term prostate cancer survivors in Ontario, Canada was identified from the Ontario Cancer Registry and contacted through their referring physician 585 prostate cancer patients, mean age 72.6 years (range 43–98) 2–13 years post diagnosis. **80 male patients (13,7%) > 80 years**	**PORPUS** **HUI2/3** **FACT-P** **PCI**	Mean utility scores were as follows: PORPUS-U = 0.92, HUI2 = 0.85, and HUI3 = 0.78. Mean health profile scores were as follows: PORPUS-*P* = 71.7, PCI sexual, urinary, and bowel function = 23.7, 79.1, and 84.6, respectively (0 = worst, 100 = best), and FACT-*P* = 125.1 (0 = worst, 156 = best). In multiple regression analyses, comorbidity, and PCI urinary, sexual, and bowel function were significant predictors of other QOL measures. With all variables, 32–50% of the variance in utilities was explained There were monotonic declines in quality of life for each decade of age (Fig. 2). For example, mean PORPUS-U scores were 0.95 in patients aged under 60 years old and 0.89 in those aged over 80 years old. Prostate Cancer (PC)-specific quality of life also declined with age. Only urinary function scores were not affected by age (Fig. 3). Global and PC-specific health status also declined across comorbidity strata (Figs.2,3) Comorbidity was the most consistent patient-related predictor of quality of life (Table3)	Many variables affect global QoL of PC survivors; only prostate symptoms and comorbidity have independent effects. Our model allows estimation of the effects of multiple factors on utilities. These utilities for long-term outcomes of PC and its treatment are valuable for decision/cost-effectiveness models of PC treatment. Prostate cancer (PC) exerts enormous health and economic burdens which will only rise in coming decades given ageing demographics and trends in Prostate Cancer diagnosis, treatment, and survivorship. This study provides unique information about the effects of PC symptoms in long-term survivors	6
2	Esbezen et al., 2004, Denmark [24]	To investigate quality of life (QoL) in elderly persons newly diagnosed with cancer (65 + years) in relation to age, contact with the health-care system, ability to perform activities of daily living (ADL), hope, social network, and support, and to identify which factors were associated with low QoL	Prospective cohort study	The sample consisted of 101 patients (75 women and 26 men) newly diagnosed with cancer. Median age was 74.74 years (IQR 8.75). **23 patients (23%) > 80 years**	**EORTC QLQ-C30**	The analysis was carried out across four age groups and revealed no significant differences among the four age groups in the three sub-scales in EORTC QLQ-C30 (Table 1). Factors significantly associated with low QoL in global health status/QoL were ‘No other incomes than retirement pension’, ‘Low level of hope’ and ‘Lung cancer’ (Table 4). Compared with the other age groups, those of a high age (80 + years) more often lived alone, used more at-home care service, and had a smaller social network. In addition, ‘being told that the cancer disease has not come to an end’, ‘needing more help in activities of daily living’, ‘getting help from grown-up children’ and ‘needing help with PADL’ were associated with low QoL Those at risk of inferior QoL, that is, being in a poor financial situation, having a low level of hope and lung cancer need special attention and specific interventions to improve QoL There were no significant differences among age groups regarding dependency in IADL and PADL (Table 2) The oldest age group (80 + years) had significantly (*P* = 0.001) fewer stays in hospital within the preceding 6 months (73.9%) and received more at-home care than the other three age groups (Table 3). There were no differences among age groups in ‘need [of] more help’ and 75.8% of the total group needed more help in daily living	The results from this study indicate that the oldest age group (80 + years) had a poorer social network and needed more support from at-home care services than the younger participants. Dependency, getting help from grown-up children and receiving help in daily living were also related to low QoL for the total sample The results also showed that the type of cancer diagnosis and perceived seriousness of it were of importance to QoL. Additionally, limited financial resources and low level of hope for an elderly person newly diagnosed with cancer were associated with a higher risk for low QoL	6
3	Thome, B. and Hallberg, I. R. (2004), Sweden [25]	To investigate quality of life (QoL) and its association with sense of coherence (SOC), complaints, comorbidity, social resources, perceived financial situation and receiving help for daily living, investigating differences between women and men aged 75 and above with cancer, and comparing women and men aged 75 and above without cancer. A further aim was to identify which of these factors was associated with low QoL in older people with cancer	Cross-sectional	Women (*n* = 74, mean age 84.3 (SD 5.9)) and men (*n* = 76, mean age 84.3 (SD 5.6)), **with a cancer disease** (*n* = 150). **93 patients (62%) > 80 years** And a matched group (age and receiving help for daily living) of women (*n* = 64, mean age 83.1) and men (*n* = 74, mean age 83.5) **without cancer** (*n* = 138)	**EORTC** **QLQ- C30** **and** **SF-12**	The only significant difference in EORTC QLQ-C30 between age groups within the study group was found in physical functioning between the youngest and the oldest age group. There were significant differences within the comparison group between age groups in the EORTC QLQ-C30 domains physical, role, cognitive and social functioning, and in symptom scales/items fatigue and appetite loss with the lowest/highest scores in the oldest age group. The study group had significantly lower scores (poorer QoL) in SF-12 (PCS, MCS) than the comparison group. However, significant differences were only found between the youngest age groups (75–79). No differences were found in SF-12 (PCS, MCS) between age groups within the study group. The comparison group, however, showed significant differences between all age groups in SF-12 PCS, with the lowest scores in the oldest age group. Women with cancer were more vulnerable than their male counterparts in QoL, SOC, perceived economic situation and social resources. Factors associated with low QoL in older people with cancer were receiving help for daily living, comorbidity, degree of complaints and pain	Younger old people seem to be more affected when stricken by cancer, whilst cancer in older age appears to be more integrated in age-associated conditions such as declining functional ability, increasing complaints and comorbidity. Receiving help for daily life contributes strongly to low QoL. Furthermore, cancer seems to affect QoL in women more than in men, and economic problems seem to be additionally related to the poorer QoL in women. Providing high-quality care to the younger segment of elderly people with cancer may require a focus on the disease, while care for the eldest may require a more comprehensive assessment of age-associated conditions as well as cancer	6

*Abbreviations*: *EORTC *QLQ-C30 European Organization for Research and Treatment of Cancer Quality of Life Questionnaire Core 30, SF-12 Short Form, *FACT-P* Functional Assessment of Cancer Therapy- Prostate, *PCI* the Prostate Cancer Index (PCI), PORPUS Patient Oriented Prostate Utility Scale, HUI2/3 The Health Utilities Index (HUI2/3)

a) Critical assessment of the studies was graded according to design specific CASP checklists https://casp-uk.net/casp-tools-checklists/, graded 1 point for “Yes", 0.5 points for “Can’t tell” (unsure), and 0 points for “No” according to the guideline by Butler et al. (2016)

### Study selection

We pooled the search results from the 8 databases. The review group screened 4,419 titles and abstracts according to the predefined set of inclusion criteria, found 8 eligible studies, screened them in full text and excluded 5 of these. The reason for exclusion at this stage was that home-dwelling cancer patients aged 80 and older were not included and that QoL was not measured. Despite extended research, it was not possible to retrieve one of the studies included in full text (Appendix 1).

### Characteristics of the included studies

The studies analyzing QoL outcomes in the subgroup of patients aged 80 years and older were Krahn et al. [23], Esbezen et al. [24] and Thome et al. [25]. The three studies were conducted in Canada, Denmark, and Sweden, respectively, and included 833 participants with various cancer diagnoses. 193 of the participants included in the review were aged 80 years or older. All studies provided information on participants’ age, ranging from 43 to 98 years, and all studies stated how many participants were aged over 80 years old (14%, 23% and 62% respectively). The sample sizes ranged from 101 to 585. The studies used prospective cohort (*n* = 2) and cross-sectional (*n* = 1) study designs. One of the studies included matched controls [25] (Table 1).

### Please insert Table 1 here

#### Methodological quality of the included studies

All three studies had a medium methodological quality and scored at least 6 of 12 possible points (Table 1, last column). They presented a focused objective and recruited patients in an acceptable manner. The main methodological drawbacks of the studies were related to question 5 on confounding factors, and question 8 on the missing confidence interval estimates of the QoL outcomes, creating difficulty in drawing conclusions on the validity of the results.

### Instruments used to measure QoL/HRQoL

QoL instruments are categorized as either disease-specific, generic or overall [26]. All the included studies used disease-specific instruments to measure QoL in cancer patients. Esbezen et al. [24] used the European Organization for Research and Treatment of Cancer Quality of Life Common 30 questionnaire (EORTC QLQ-C30) [27] and Thome et al. [25] used both the EORTC QLQ-C30 [27] and the 12-Item Short Form Health Survey (SF-12) [28]. A generic utility instrument, the Health Utilities Index (HUI2/3) [29], as well as a diagnosis-specific QoL instrument, the Functional Assessment of Cancer Therapy- Prostate (FACT-P) [30] was included in Krahn et al. [23]. Additionally, the Prostate Cancer Index (PCI) [31] and the Patient Oriented Prostate Utility Scale (PORPUS) [32], was used as both a health profile instrument (PORPUS-P) and a utility instrument (PORPUS-U) [32–34].

### Quality of life in home-dwelling cancer patients aged 80 years or older

In Krahn et al. [23], they aimed to measure quality of life (QoL) and utilities for prostate cancer (PC) patients and determine their predictors. The study found that there were monotonic declines in quality of life for each decade of age. For example, mean PORPUS-U scores were 0.95 in patients aged below 60 years and 0.89 in those aged above 80 years. PC-specific quality of life also declined with age. Only urinary function scores were not affected by age. Global and PC-specific health status also declined across comorbidity strata. Krahn et al. [23] conclude that many variables affect global QoL of Prostate Cancer survivors, but only prostate symptoms and comorbidity have independent effects. Comorbidity was the most consistent patient-related predictor of quality of life.

Esbezen et al. [24] investigated QoL in elderly persons newly diagnosed with cancer (65 + years) in relation to age, contact with the health-care system, ability to perform activities of daily living (ADL), hope, social network and support. The analysis was carried out across four age groups and revealed no significant differences among the four age groups in the three subscales of global health status/QoL, functional scale and symptom scale in EORTC QLQ-C30. Factors significantly associated with low QoL in global health status/QoL were ‘No other incomes than retirement pension’, ‘Low level of hope’ and ‘Lung cancer’. Compared with the other age groups, those of a higher age (80 + years) more often lived alone, used more at-home care service, and had a smaller social network. In addition, ‘being told that the cancer disease has not come to an end’, ‘needing more help in activities of daily living’, ‘getting help from grown-up children’ and ‘needing help with personal activities of daily living (PADL)’ were associated with low QoL.

Thome et al. [25] investigated QoL and its associations with sense of coherence, complaints, comorbidity, social resources, perceived financial situation and receiving help from others for daily living. They investigated differences between women and men aged 75 and above with cancer, and compared this to women and men aged 75 and above without cancer. A further aim was to identify which of these factors were associated with low QoL in older people with cancer. When studying all five domains of the EORTC QLQ-C30, the only significant difference between age groups within the study group was found in physical functioning between the youngest (75–79) and the oldest (80–91) age groups. The study group had significantly lower scores (poorer QoL) in SF-12 than the comparison group. However, significant differences were only found between the youngest age groups (75–79). No differences were found in SF-12 between age groups within the study group. The comparison group, however, showed significant differences between all age groups in SF-12 and physical component score (PCS), with the lowest scores being found in the oldest age group. The authors conclude that women with cancer were more vulnerable than their male counterparts in QoL, SOC, perceived financial situation and social resources. Factors associated with low QoL in older people with cancer were receiving help for daily living, comorbidity, degree of complaints and pain. Cancer in older age appears to be more integrated in age-associated conditions such as declining functional ability, increasing complaints and comorbidity. Furthermore, cancer seems to affect QoL in women more than in men, and financial issues seem to be more related to the poorer QoL in women [25].

## Discussion

### Quality of life in home-dwelling cancer patients aged 80 years or older

Our systematic search yielded more than 4,000 articles, most of them including home-dwelling cancer patients aged 80 years and older in their cohorts. Interestingly, only three studies conducted subgroup analyses by age, thus generating very limited evidence of QoL in the subgroup of 80 years and older and limited results for our synthesis. This finding represents a paradox as the population of elderly cancer patients aged over 80 years old is increasingly growing. The cumulative impact of old age, cancer and multimorbidity is important to study in relation to QoL, as older adults often lack the physiological reserves required to effectively recover from cancer treatment. In turn, this may lead to problems related to QoL, encompassing physical, emotional, and social functioning [10, 11]. The concept of quality of life is multidimensional, and includes several different domains [11]. The relevance of a specific domain may vary according to stage and type of illness, age, and cultural background [35]. The “COSMIN Study Design checklist for Patient-reported outcome measurement instruments” [17] recommends using age- and diagnosis- specific instruments, such as the EORTC- QLQ- ELD15 [36] in regard the specific needs of aged cancer patients. None of the included studies in this review had used an age specific QoL instrument.

Krahn et al. [23] found monotonic declines in QoL for each decade of age in a geographically diverse sample of long-term prostate cancer survivors in Ontario, Canada. Comorbidity was the most consistent patient-related predictor of QoL [23]. As this is one specific diagnosis group, the results cannot be directly applied to other cancer patient groups, but cancer patients over the age of 80 often have multiple health issues and comorbidities, and therefore cancer treatment can be more challenging and complex [5, 6].

Cancer patients over the age of 80 represent a population who are vulnerable and frail [6]. Gessink et al. [13] found that frailty is associated with comorbidity and that dealing with cancer was associated with lower QoL in older patients *under* the age of 80. They furthermore found that there was a significant association between increasing frailty and lower QoL [13]. This may indicate that cancer patients *over* the age of 80, being even more frail and vulnerable, are particularly exposed to declining QoL. Frailty in geriatric oncology reports has mainly been evaluated in relation to patients’ ability to tolerate cancer treatment, mostly taking morbidity and survival into consideration, not a direct relationship with QoL [13].

Thome et al. [25] found in a study of women and men aged 75 and above with cancer, and a matched group without cancer that the only significant difference in EORTC QLQ-C30 between age groups within the study group was in physical functioning between the youngest (75–79) and the oldest (80–91) age group. No differences were found in SF-12 between age groups within the study group. Furthermore, older people under the age of 79 seem to be more affected when stricken by cancer, whilst cancer in patients over 80 years old seems to be more integrated with age-associated conditions such as declining functional ability, increasing complaints and comorbidity [25]. This may be explained by the increase of comorbidity and frailty at more advanced ages and a change in life expectations when getting older.

When studying elderly persons newly diagnosed with cancer (65 + years) in relation to age, contact with the health-care system, ability to perform activities of daily living (ADL), hope, social network, support, and QoL, Esbenzen et al. [24] found no significant differences among the four age groups in the three subscales studied in EORTC QLQ-C30. Factors significantly associated with low QoL in global health status/QoL were ‘No other incomes than retirement pension’ and ‘Low level of hope’ [24]. Low income and financial difficulties are associated with a significantly worse overall clinical HRQoL [37]. Esbezen’s findings on QoL must be seen in conjunction with the significant differences among age groups regarding dependency in instrumental activities of daily living (IADL) and PADL [24]. The oldest age group (80 + years) had significantly (*P* = 0.001) fewer stays in hospital within the preceding 6 months (73.9%) and received more at-home care than the other three age groups [24]. There were no differences among age groups in ‘need [of] more help’ and 75.8% of the total group needed more help in their daily lives. The results from this study indicate that the oldest age group (80 + years) had a poorer social network and needed more support from at-home care services than the younger participants.

Among older home-dwelling cancer patients, women report significantly higher scores of anxiety and depression than men [13]. Women also experience higher emotional distress than men [14]. Solvik et al.’s study on pain, fatigue, anxiety and depression in older home‐dwelling people with cancer reports a significant difference between men and women in terms of civil status, ongoing treatment, anxiety and depression. In their study there were more single women and more women who underwent treatment than men [14]. Also, women participating in studies are older than men [5, 6]. As frailty increases with age, this could explain the higher scores reported by the women. Economic issues, pain, and other symptoms, such as anxiety and depression and being dependent on help from others may impact QoL [9, 13, 14]. The oldest age group (80 + years) had significantly (*P* = 0.001) fewer stays in hospital within the preceding 6 months (73.9%), although the oldest age group also had a poorer social network and needed more support from at-home care services [24]. Having fewer stays in hospital in spite of poorer social support is interesting and should be examined further. One explanation may be that having a poorer social network increases the need of at-home care services, which may secure a more pro-active provision of at-home care and therefore prevent hospital admission.

Receiving help in everyday life contributed strongly to low QoL in Thome et al.’s [25] study. Furthermore, women with cancer were more vulnerable than their male counterparts in terms of QoL. It is up for debate whether needing help in everyday life is associated with a lower QoL, or if a lower QoL is responsible for the increased need of at-home care services. This, and why women are more vulnerable in terms of QoL, should be investigated further. Other factors associated with low QoL in older people with cancer besides receiving help for their everyday lives, were comorbidity, degree of complaints and pain [19, 20]. This indicates that assessment of symptoms and adequate symptom treatment could prevent a decrease in QoL. There seem to be differences in the assessment of symptoms and QoL between patients and health care workers in home care settings, this includes anxiety level, personal thoughts, practical matters, and information received [10], indicating the need for regular patient self-assessment of symptoms and QoL.

Thome et al. [25] state that cancer in older age appears to be more integrated in age-associated conditions such as declining functional ability, increasing complaints and comorbidity, indicating that providing high-quality care to the younger segment of elderly people with cancer may require a focus on the disease, while care for the oldest may require a more comprehensive assessment of age associated conditions as well as cancer. This implies a need for an age specific approach in the health care services, and Berntsen et al. [38] argue that using an individual-oriented and pro-active approach when caring for multimorbid elderly can reduce the risk of high-level emergency care, increase use of low-level planned care, and substantially reduce mortality risk.

### QoL Instruments used

The included studies used both generic and disease-specific QoL instruments. The EORTC QLQ-C30 [27] was employed by both Esbezen [24] and Thome et al. [25]. Thome additionally used the generic 12-Item Short Form Health Survey (SF-12) [28]. Only Krahn et al. [23] used diagnosis-specific instruments, FACT-P, PCI, PORPUS [23]. None of the included studies used instruments specifically designed and approved for elderly cancer patients. Taking into consideration the complexity and multidimensionality of older cancer patients’ situation [5–7], this represents a limitation of all the included studies. Multidimensional and age-specific scales such as the EORTC-QLQ-ELD [36,14] should be used to secure valid and reliable information on all the dimensions of QoL to provide a better understanding of how the elderly patients experience their QoL.

None of the QoL instruments used in the three studies include spirituality as a core domain. Earlier research suggests that categories that include spirituality, such as meaning and hope, might be important aspects of QoL that may be particularly important in the context of life-threatening illness such as cancer [39]. In line with Padilla et al.’s definition of health- related QoL [40], spiritual well-being should be evaluated “*at a point in time when health, illness, and treatment conditions are relevant*”. The perception of Quality of life is subjective, and therefore, patients' viewpoints may substantially differ from the judgement of physicians. The relevance of a specific domain may vary according to stage and type of illness, age, social support, spiritual preferences and cultural background. If it is universally accepted that patients should measure their own quality of life, then patients themselves should also select what to measure, and weight the relevance of each domain and subdomain included in a QoL instrument [41]. This is in line with the “COSMIN Study Design checklist for Patient-reported outcome measurement instruments” [17].

### Methodologically limitations

Several methodological issues limit the conclusions of this review. Our systematic review was restricted to the English language, potentially introducing a language bias, and other studies may have been missed. The fact that we only succeeded in finding three studies that performed subgroup analysis of QoL in home-dwelling cancer patients aged 80 years or older is the strongest limitation.

The three included studies differed in many respects, such as design, population, sample size, age range, aim, instruments used and outcome, thus making it impossible to make a meta-analysis, as well as compromising the ability to provide a clear and unambiguous conclusion. Furthermore, none of the studies applied an age specific instrument, yielding a validity challenge. The medium methodical quality of the included studies also represents a limitation. Especially considering confounding factors in one of the studies [25], and the missing confidence interval of the QoL outcomes in all three studies [23–25]. Therefore, the validity of the QoL results cannot be conclusive. This further implies that the results must be interpreted with caution.

## Conclusion

Despite heterogeneity in the three included studies when it comes to cancer diagnoses, instruments used, and outcomes studied, QoL in home-dwelling cancer patients aged over 80 years old seems to be correlated with age, physical functioning, comorbidity, living alone, needing at-home care services, having a poor financial situation and a small social network. Only one of the included studies used a diagnosis-specific instrument, and none of the studies used an age specific instrument. Our findings reveal a gap in the knowledge base of this increasingly challenging patient group. The lack of evidence in this population may negatively impact healthcare personnel’s practice and priorities when offering care for home-dwelling cancer patients aged over 80 years old. Based on this finding, we strongly recommend future studies to include cancer patients aged over 80 years old, and to perform subgroup analyses based on age. In addition to using generic instruments, valid age and diagnosis-specific QoL instruments must be applied.

## Supplementary Information

**Additional file 1.**

## Data Availability

Not applicable.

## Abbreviations

ADL: Activities of daily living
CASP: Critical Appraisal Skills Programme
EORTC QLQ-C30: European Organization for Research and Treatment of Cancer Quality of Life Questionnaire Core 30
FACT-P: Functional Assessment of Cancer Therapy- Prostate
HUI2/3: The Health Utilities Index
HRQoL: Health related quality of life
IADL: Instrumental activities of daily living
PADL: Physical activities of daily living
PC: Prostate cancer
PCI: The Prostate Cancer Index
PORPUS: Patient Oriented Prostate Utility Scale
QoL: Quality of life
SF-12: Short Form Health Survey 12 questions
SOC: Sense of coherence
