# Supplementary material for: Quality of life in home-dwelling cancer patients aged 80 years and older: a systematic review
Source: Health Qual Life Outcomes. 2022 Nov 28;20:154. doi: 10.1186/s12955-022-02070-1 (PMC9703757; doi:10.1186/s12955-022-02070-1)
Supplement: Supplementary file 1 — Additional file 1. [file 12955_2022_2070_MOESM1_ESM.docx]

**Appendix 1. Search strategy**

Quality of life in community dwelling cancer patients 80 years and older: a systematic review

Inger Helen Hardeland Hjelmeland, Faculty of Health and Social Sciences, Western Norway University of Applied Sciences, Stord, Norway and VID Specialized University, Ulriksdal 10, 5009 Bergen, Norway

Jorunn Drageset, Faculty of Health and Social Sciences, Western Norway University of Applied Sciences, Inndalsveien 28, 5063 Kronstad, Bergen, Norway and Department of Global Health and Primary Care, University of Bergen, Postboks 7804, 5020 Bergen, Norway

Øyvind Nordvik, Faculty of Health and Social Sciences, Western Norway University of Applied Sciences, Bergen, Norway, Inndalsveien 28, 5063 Kronstad, Bergen, Norway

Elisabeth Grov Beisland, Faculty of Health and Social Sciences, Western Norway University of Applied Sciences, Bergen, Norway, Inndalsveien 28, 5063 Kronstad, Bergen, Norway

Content

[Description of the search 1](#_Toc117587252)

[Cochrane 2](#_Toc117587253)

[Epistemonikos 2](#_Toc117587254)

[Campbell? 3](#_Toc117587255)

[Medline (Ovid) 3](#_Toc117587256)

[EMBASE 3](#_Toc117587257)

[CINAHL 4](#_Toc117587258)

[PsycINFO 5](#_Toc117587259)

[Scopus 5](#_Toc117587260)

[Duplicates 6](#_Toc117587261)

Description of the search

**Focus 1:**

Neoplasms

Medical Oncology

**Keywords:**

Cancer* OR neoplas* OR oncolog* OR malign* OR tumor* OR tumour* OR carsinoma* OR metasta*

**Focus 2:**

Aged 80 years and over

Aged

Frail elderly

**Keywords:**

aged or elder* or aging or ageing or old* OR geriatric* OR octogeneri* OR nonageneri* OR centenari*

**Focus 3:**

Independent living/

Housing

Housing for the elderly

**Keywords:**

"Community dwelling" OR "Community living" OR "Home dwelling" OR (Living W2 home) OR (living W2 independent*) OR Housing OR Homebound OR Lodging* OR "Senior housing"

Cochrane


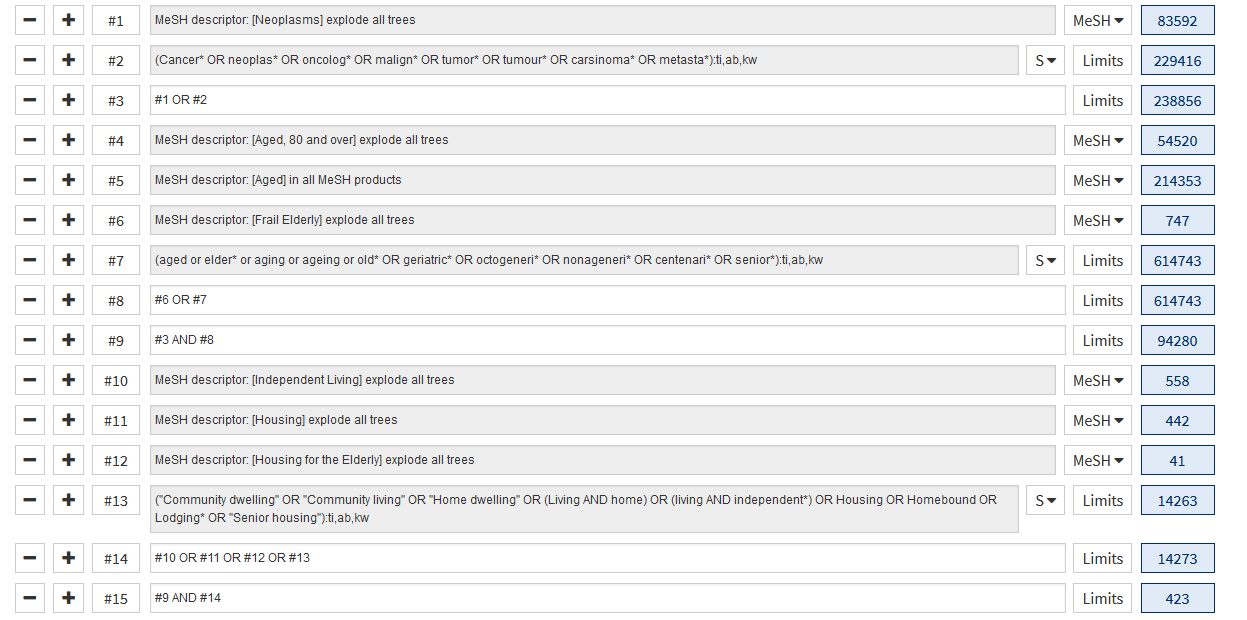


This search yealded 423 hits, of which 16 systematic reviews, 0 protocols, and 407 primary clinical studies.

Epistemonikos

(title:((title:((title:(Cancer* OR neoplas* OR oncolog* OR malign* OR tumor* OR tumour* OR carsinoma* OR metasta*) OR abstract:(Cancer* OR neoplas* OR oncolog* OR malign* OR tumor* OR tumour* OR carsinoma* OR metasta*))) OR abstract:((title:(Cancer* OR neoplas* OR oncolog* OR malign* OR tumor* OR tumour* OR carsinoma* OR metasta*) OR abstract:(Cancer* OR neoplas* OR oncolog* OR malign* OR tumor* OR tumour* OR carsinoma* OR metasta*)))) AND (title:(aged OR elder* OR aging OR ageing OR old* OR geriatric* OR octogeneri* OR nonageneri* OR centenari* OR senior*) OR abstract:(aged OR elder* OR aging OR ageing OR old* OR geriatric* OR octogeneri* OR nonageneri* OR centenari* OR senior*))) OR abstract:((title:((title:(Cancer* OR neoplas* OR oncolog* OR malign* OR tumor* OR tumour* OR carsinoma* OR metasta*) OR abstract:(Cancer* OR neoplas* OR oncolog* OR malign* OR tumor* OR tumour* OR carsinoma* OR metasta*))) OR abstract:((title:(Cancer* OR neoplas* OR oncolog* OR malign* OR tumor* OR tumour* OR carsinoma* OR metasta*) OR abstract:(Cancer* OR neoplas* OR oncolog* OR malign* OR tumor* OR tumour* OR carsinoma* OR metasta*)))) AND (title:(aged OR elder* OR aging OR ageing OR old* OR geriatric* OR octogeneri* OR nonageneri* OR centenari* OR senior*) OR abstract:(aged OR elder* OR aging OR ageing OR old* OR geriatric* OR octogeneri* OR nonageneri* OR centenari* OR senior*)))) AND (title:("Community dwelling" OR "Community living" OR "Home dwelling" OR (Living AND home) OR (living AND independent*) OR Housing OR Homebound OR Lodging* OR "Senior housing") OR abstract:("Community dwelling" OR "Community living" OR "Home dwelling" OR (Living AND home) OR (living AND independent*) OR Housing OR Homebound OR Lodging* OR "Senior housing"))

Hits: Systematic reviews: 20, Broad synthesis: 1, Primary studies: 108

Campbell

Hits: 0.

Medline (Ovid)

Ovid MEDLINE(R) and Epub Ahead of Print, In-Process, In-Data-Review & Other Non-Indexed Citations and Daily <1946 to September 20, 2021>

1 exp neoplasms/ 3537345

2 (Cancer* or neoplas* or oncolog* or malign* or tumor* or tumour* or carsinoma* or metasta*).ab,kf,ti. 3544792

3 1 or 2 4627410

4 aged/ or "aged, 80 and over"/ or frail elderly/ 3302878

5 (aged or elder* or aging or ageing or old* or geriatric* or octogeneri* or nonageneri* or centenari* or senior*).ab,kf,ti. 2415141

6 4 or 5 5012858

7 Independent Living/ 8299

8 housing/ or housing for the elderly/ 20301

9 ("Community dwelling" or "Community living" or "Home dwelling" or (Living adj2 home) or (living adj2 independent*) or Housing or Homebound or Lodging* or "Senior housing").ab,kf,ti. 71626

10 7 or 8 or 9 86993

11 3 and 6 and 10 1666

Hits: 1666.

EMBASE

Embase <1974 to 2021 Week 37>

1 exp neoplasm/ 4805623

2 (Cancer* or neoplas* or oncolog* or malign* or tumor* or tumour* or carsinoma* or metasta*).ab,kf,ti. 4686343

3 1 or 2 5934941

4 aged/ or frail elderly/ or very elderly/ 3226557

5 (aged or elder* or aging or ageing or old* or geriatric* or octogeneri* or nonageneri* or centenari* or senior*).ab,kf,ti. 3331558

6 4 or 5 5640210

7 independent living/ 5651

8 ("Community dwelling" or "Community living" or "Home dwelling" or (Living adj2 home) or (living adj2 independent*) or Housing or Homebound or Lodging* or "Senior housing").ab,kf,ti. 87883

9 7 or 8 89635

10 3 and 6 and 9 2316

Hits: 2316.

CINAHL

| Tuesday, September 21, 2021 11:39:13 AM |
| --- |

| **#** | **Query** | **Results** |
| --- | --- | --- |
| S11 | S3 AND S6 AND S10 | 964 |
| S10 | S7 OR S8 OR S9 | 58,622 |
| S9 | TI ( "Community dwelling" OR "Community living" OR "Home dwelling" OR (Living N2 home) OR (living N2 independent*) OR Housing OR Homebound OR Lodging* OR "Senior housing" ) OR AB ( "Community dwelling" OR "Community living" OR "Home dwelling" OR (Living N2 home) OR (living N2 independent*) OR Housing OR Homebound OR Lodging* OR "Senior housing" ) OR SU ( "Community dwelling" OR "Community living" OR "Home dwelling" OR (Living N2 home) OR (living N2 independent*) OR Housing OR Homebound OR Lodging* OR "Senior housing" ) | 54,446 |
| S8 | (MH "Housing for the Elderly") OR (MH "Housing") | 12,668 |
| S7 | (MH "Community Living") OR (MH "Assisted Living") OR (MH "Community Reintegration") | 21,985 |
| S6 | S4 OR S5 | 1,235,663 |
| S5 | TI ( aged or elder* or aging or ageing or old* OR geriatric* OR octogeneri* OR nonageneri* OR centenari* OR senior* ) OR AB ( aged or elder* or aging or ageing or old* OR geriatric* OR octogeneri* OR nonageneri* OR centenari* OR senior* ) OR SU ( aged or elder* or aging or ageing or old* OR geriatric* OR octogeneri* OR nonageneri* OR centenari* OR senior* ) | 1,235,663 |
| S4 | (MH "Aged") OR (MH "Aged, 80 and Over") OR (MH "Frail Elderly") | 880,340 |
| S3 | S1 OR S2 | 828,532 |
| S2 | TI ( Cancer* OR neoplas* OR oncolog* OR malign* OR tumor* OR tumour* OR carsinoma* OR metasta* ) OR AB ( Cancer* OR neoplas* OR oncolog* OR malign* OR tumor* OR tumour* OR carsinoma* OR metasta* ) OR SU ( Cancer* OR neoplas* OR oncolog* OR malign* OR tumor* OR tumour* OR carsinoma* OR metasta* ) | 772,443 |
| S1 | (MH "Neoplasms+") | 591,927 |

Hits: 964.

PsycINFO

APA PsycInfo <1987 to September Week 2 2021>

1 exp neoplasms/ 53366

2 (Cancer* or neoplas* or oncolog* or malign* or tumor* or tumour* or carsinoma* or metasta*).ab,ti,tw. 84727

3 1 or 2 87226

4 geriatric patients/ 12141

5 older adulthood/ 5613

6 (aged or elder* or aging or ageing or old* or geriatric* or octogeneri* or nonageneri* or centenari* or senior*).ab,ti,tw. 644098

7 4 or 5 or 6 644644

8 "activities of daily living"/ or aging in place/ or independent living programs/ 6871

9 ("Community dwelling" or "Community living" or "Home dwelling" or (Living adj2 home) or (living adj2 independent*) or Housing or Homebound or Lodging* or "Senior housing").ab,ti,tw. 34873

10 8 or 9 40529

11 3 and 7 and 10 345

Hits: 345 .

Scopus

( TITLE-ABS-KEY ( *"Community dwelling"*  OR  *"Community living"*  OR  *"Home dwelling"*  OR  ( *living*  W/2  *home* )  OR  ( *living*  W/2  *independent** )  OR  *housing*  OR  *homebound*  OR  *lodging**  OR  *"Senior housing"* )  AND  TITLE-ABS-KEY ( *cancer**  OR  *neoplas**  OR  *oncolog**  OR  *malign**  OR  *tumor**  OR  *tumour**  OR  *carsinoma**  OR  *metasta** )  AND  TITLE-ABS-KEY ( *aged*  OR  *elder**  OR  *aging*  OR  *ageing*  OR  *old**  OR  *geriatric**  OR  *octogeneri**  OR  *nonageneri**  OR  *centenari**  OR  *senior** ) )

Hits: 2654.

Duplicates

When all references were imported to an EndNote library there was all together 12 722 references in the library. After the first remove of duplicates both using EndNote and manually, 8497 references remained. There were still some duplicates that needed to be assessed in more detail.(e.g. same authors, title and sidenumbers, but different year). 8497 references were imported to an EndNote-base. 4078 of these references were duplicates. Removing of duplicates resulted in a total of 4419 references.

**Appendix 2.** **PRISMA 2020 flow diagram**

**Identification of studies via databases and registers**

Records identified from*:

Databases

Medline(n=1666)

Cohcrane(n= 423)

EMBASE(n=2316)

Epistemonikos(n=129)

Campell(n=0)

CINAHL(n=964)

PsycINFO(n=345)

Scopus(n=2654)

(n = 8497)

Registers (n = )

Records removed *before screening*:

Duplicate records removed (n = 4078)

Records marked as ineligible by automation tools (n = 0)

Records removed for other reasons (n =0 )

**Identification**

Records screened

(n = 4419)

Records excluded**

(n =4413 )

Reports sought for retrieval

(n =1 )

Reports not retrieved

(n =1 )

**Screening**

Reports excluded: (n=5)

Reason 1 (n =1 )

Reason 2 (n = 4)

Reports assessed for eligibility

(n = 8)

Studies included in review

(n = 3)

**Included**

Reason 1: Wrong outcome

Reason 2: Wrong population group

*From:*  Page MJ, McKenzie JE, Bossuyt PM, Boutron I, Hoffmann TC, Mulrow CD, et al. The PRISMA 2020 statement: an updated guideline for reporting systematic reviews. BMJ 2021;372:n71. doi: 10.1136/bmj.n71

For more information, visit: <http://www.prisma-statement.org/>
